# Interepidemic Rift Valley Fever Virus Seropositivity, Northeastern Kenya

**DOI:** 10.3201/eid1408.080082

**Published:** 2008-08

**Authors:** A. Desiree LaBeaud, Eric M. Muchiri, Malik Ndzovu, Mariam T. Mwanje, Samuel Muiruri, Clarence J. Peters, Charles H. King

**Affiliations:** * Rainbow Babies and Children’s Hospital, Cleveland, Ohio, USA; † Case Western Reserve University Center for Global Health and Diseases, Cleveland; ‡ Ministry of Health, Nairobi, Kenya; § University of Texas Medical Branch, Galveston, Texas, USA

**Keywords:** Rift Valley fever, Kenya, hemorrhagic fevers/viral, bioterrorism, arboviruses, Bunyaviridae, risk factors, epidemiology, research

## Abstract

Most outbreaks of Rift Valley fever (RVF) occur in remote locations after floods. To determine environmental risk factors and long-term sequelae of human RVF, we examined rates of previous Rift Valley fever virus (RVFV) exposure by age and location during an interepidemic period in 2006. In a randomized household cluster survey in 2 areas of Ijara District, Kenya, we examined 248 residents of 2 sublocations, Gumarey (village) and Sogan-Godud (town). Overall, the RVFV seropositivity rate was 13% according to immunoglobulin G ELISA; evidence of interepidemic RVFV transmission was detected. Increased seropositivity was found among older persons, those who were male, those who lived in the rural village (Gumarey), and those who had disposed of animal abortus. Rural Gumarey reported more mosquito and animal exposure than Sogan-Godud. Seropositive persons were more likely to have visual impairment and retinal lesions; other physical findings did not differ.

## Abstract 2

Exposure is associated with long-term retinal disease and is most common in rural settings among older men who have contact with aborting animals.

Rift Valley fever (RVF) is a mosquito-borne zoonosis that is expanding its range in Africa and the Middle East. Economic effects can be catastrophic for meat and dairy producers, e.g., high illness and mortality rates among affected livestock herds (*1*,*2*) prompting World Organization for Animal Health–mandated international embargoes of livestock exports. These epidemics are even more devastating for pastoral nomads and local herders; many adult animals can die, affecting the next crop of newborns and the survival of locals who are economically and physically dependent on milk and meat during the epidemic. During large RVF outbreaks, extensive numbers of human infections occur as well, leading to substantial healthcare challenges in resource-limited settings. RVF symptoms in persons are typically fever, myalgia, and malaise; in a noteworthy minority of cases retinitis, encephalitis, hemorrhagic fever, and death occur. Overall mortality rate is ≈1% (*3*,*4*).

RVF is caused by the phlebovirus, Rift Valley fever virus (RVFV), which was originally isolated in Kenya and is endemic to other countries of East Africa, South Africa, and the Senegal River valley (*3*,*5*–*7*). The virus, introduced repeatedly into Egypt since the 1970s, and most recently into the Arabian peninsula (Yemen and Saudi Arabia) in 2000 (*8*–*10*), is embedded in ecosystems by vertical transmission in certain floodwater *Aedes* mosquito species (*1*). Consequently, RVF outbreaks are strongly linked to excessive rainfall and local flooding. The most recent Kenyan Rift Valley fever outbreak occurred during El Niño rains from November 2006 through April 2007 (*11*,*12*). The largest RVF outbreak in Kenya took place in an El Niño–related flooding period in 1997–1998 (*13*). Even within different climate zones, RVFV transmission may vary considerably as a function of fine-scale differences in local environment.

Evidence of prior RVFV infection can be tested by ELISA for anti-RVFV immunoglobulin (Ig) G (*14*,*15*). Earlier studies have shown that RVFV seroprevalence in Kenyan populations has been as high as 32% in high-risk areas during epidemics (*13*). During interepidemic periods, observed community RVFV seroprevalence rates have ranged from 1% to 19% in different settings within Kenya (*16*).

Because RVF outbreaks typically occur in remote locations under extreme weather conditions, relatively little is known about the underlying health status of at-risk communities. Likewise, debate continues regarding the likely dominant mode of animal-to-human transmission during combined epizootics and epidemics. RVFV reemergence, caused by floodwater mosquitoes, is followed by widespread amplification in high-risk animal populations and progressively greater prevalence among animals. When epizootic conditions are right, additional mosquito species will feed on viremic animals and subsequently transmit RVFV to humans, creating a potential epidemic. Humans can also become infected through exposure to infectious animal tissues or bodily fluids such as abortus, birthing fluids, milk, or blood. Among pastoral nomads and other herders in the semiarid regions of Africa, family members could be differentially exposed depending on traditional gender-specific duties, thereby altering the risk-modifying effects of age or gender. Specific types of animal exposure that are the most risky, and important nonanimal exposures have not yet been elucidated. Knowing which forms of exposure provide the greatest RVFV transmission risk may be useful for endemic or epidemic public health education and for targeting interventions (such as animal vaccination) that can decrease infection or illness during an epidemic. The goals of this study were to 1) determine the baseline human population health status in an area that has suffered repeated RVF outbreaks; 2) identify which animal and nonanimal exposures are associated with RVFV seropositivity; 3) evaluate whether seropositivity, exposures, and risks differ among town and village settings in a high-risk region of northeastern Kenya; and 4) assess whether interepidemic human RVFV transmission occurs.

## Materials and Methods

### Location

**Figure 1:**
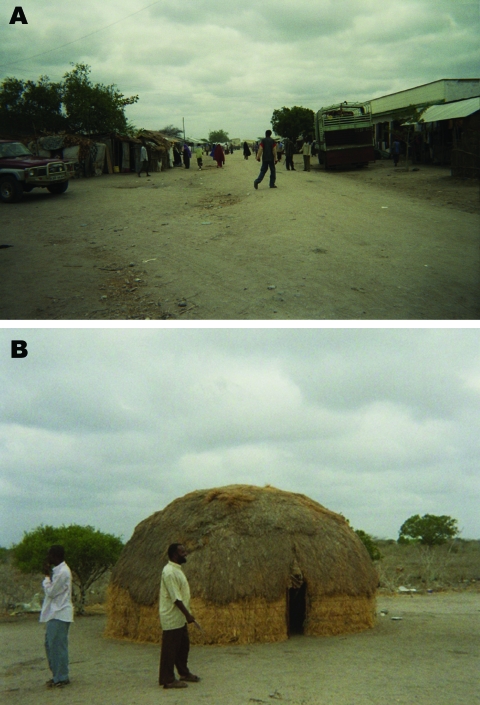
Photographs depicting differences between sublocations in northeastern Kenya. Sogan-Godud (A) has more permanent dwellings and stores with tin-roofed buildings. Gumarey (B) has more semipermanent traditional dwellings and animal grazing areas.

Our study was a location-stratified household-based cluster sampling of human populations residing in 2 areas near Masalani Town, Ijara District, situated in a semiarid region of Northeastern Province, Kenya. The study was performed in March and April 2006, ≈8.5 years after the previous RVF outbreak of 1997–1998, and well before the floods during the fall of 2006 that were associated with the most recent RVF epizootic/epidemic. On the basis of our study objectives, the balanced sampling frame for selection of the planned 250 participants was divided between a rural village, Gumarey (centered at 1° 40′12′′S, 40°10′48′′E), and a town, Sogan-Godud (centered at 1°41′24′′S, 40°10′12′′E). Both are sublocations defined within the Kenya Census and are located within 500 m of each other and within 10 km of the Tana River, which is prone to flooding during periods of excessive rainfall. Flatness of the local terrain, combined with poor drainage, makes the area a prime environment for RVFV transmission during floods, as evidenced by ongoing RVF outbreaks. Gumarey has a largely seminomadic pastoralist population, and local homes are traditional grass huts. Sogan-Godud is a larger town with more permanent tin-roofed dwellings and stores (Figure 1).

### Population

Study recruitment was begun after consultation and approval by local administrators and religious leaders. After an initial demographic census was conducted to determine the current local population and its distribution, 270 survey participants were selected by randomized cluster sampling of households in the 2 designated subsections of Masalani town. Children <1 year of age and those residing in the area <2 years were excluded. All adult participants provided informed consent. Parents provided informed consent for participating children; children >7 years of age provided individual assent. The study sample comprised a locally representative ethnic mix of >99% Somali or Bantu and <1% Indian or other Asian. Participating households were sampled by using a probability proportionate to size approach. Nonparticipating households were substituted for by using additional, randomly-selected households chosen according to sampling rules established at the outset of the survey.

### Examination Procedures

Study participants received a structured interview regarding housing, animal exposure, motor function, visual function, and recent or remote RVF-related symptoms (questionnaire in Technical Appendix 1; accompanying parents served as proxies for children when necessary). Participants also received a complete physical examination, a vision test and indirect ophthalmoscopic examination for signs of current or previous retinal inflammation, and phlebotomy (i.e., 5 mL venous blood samples from persons >5 years of age and 1 mL from children <5 years of age).

### Laboratory Testing

The primary measure of RVFV exposure was seropositivity, indicated by serum anti-RVFV IgG detection using ELISA. Specimens were screened for the presence of anti-RVFV IgG by ELISA by using lysates of Vero cells infected with the MP-12 strain (vaccine strain) of RVFV as the test antigen and lysates of mock-infected cells as the internal control antigen. This assay has been established and validated in previous survey studies (*15*,*16*). Serum samples diluted 1:100 were read at 405 nm; those with an optical density (OD) value (corrected for reactivity on normal cell antigen) > mean + 2 standard deviations for control serum and absolute value >0.2 were deemed positive. Each sample was run in duplicate, and OD values were averaged. Any OD discrepancy between duplicate tests was resolved by repeat testing. Pooled RVFV-positive serum samples were used as the positive plate controls, and pooled RVFV-negative North American serum samples were used as the negative plate controls. Serologic screening was performed at the Division of Vector-Borne Diseases in Nairobi and confirmed at Case Western Reserve University; correlation of results was excellent. Confirmatory plaque reduction neutralization test (PRNT) was performed at University of Texas Medical Branch at Galveston to assess the risk of false-positive results secondary to ELISA cross-reactivity with related viruses. Confirmatory testing using PRNT was performed on all positive samples (n = 33) and an age- and location-matched set of negative samples (n = 33) (*17*). All ELISA-positive samples had PRNT titers >80; most had titers of 320. All but 1 ELISA-negative sample had titers <10. This apparently false-negative sample had a PRNT titer of 80 on repeated testing.

### Statistical Analysis

Initial univariate analysis was conducted to describe demographic variables (Appendix Table 1) Bivariate analysis was based on χ^2^ test (or Yates correction to the χ^2^ test where appropriate) of several potential predictors of RVFV seropositivity (Appendix Table 1) as well as bivariate comparisons between villages (Appendix Table 2). After initial bivariate analysis of RVFV-seropositivity outcomes, predictor variables were further tested for association with RVFV seropositivity by using multivariable logistic regression. Data for all 248 participants were modeled by using predictor variables that had been determined by bivariate analysis to be associated with RVFV seropositivity (Technical Appendix 2). Logistic models were also constructed by village to determine local predictors of RVFV seropositivity (Appendix Table 3). Individual predictors were tested for multicolinearity by using χ^2^ test. Hosmer-Lemeshow goodness-of-fit χ^2^ values were calculated for all logistic models and indicated that model predictors sufficiently described the observed data (Technical Appendix 2 and Appendix Table 3). All bivariate analysis and logistic modeling was initially performed by using R software version 2.3.1 (www.r-project.org/index.html) and confirmed by using SPSS version 15.0 for Windows (SPSS Inc., Chicago, IL, USA).

### Ethical Considerations

This study was performed under a human research protocol approved by the Human Investigations Review Board of University Hospitals of Cleveland and the Ethical Review Committee of the Kenya Medical Research Institute. It is registered as Clinical Trial NCT00287014 and available from www.clinicaltrials.gov.

## Results

### Survey Results

A total of 270 potential participants were invited to participate; they were selected by randomized cluster sampling of 66 households in the 2 designated administrative sublocations of Masalani Town in Ijara district. Of this selected sample, 248 (91.9%) completed all study procedures, including serum testing (Appendix Table 1).

**Figure 2:**
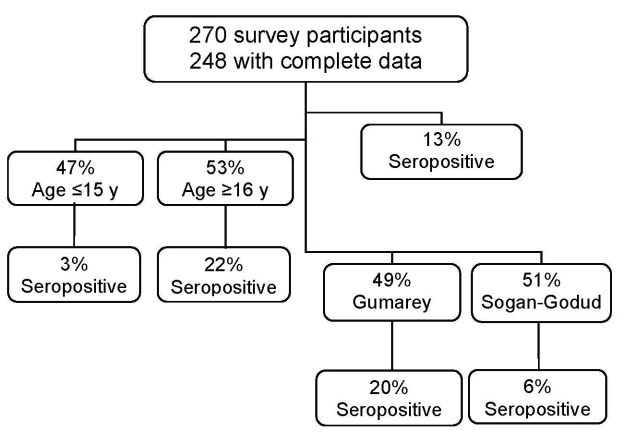
Flowchart of northeastern Kenya Rift Valley fever virus study participants.

The final study cohort comprised 248 participants, of whom 33 (13%, 95% confidence interval [CI] 9.3–18.1) were RVFV seropositive. Of the 248, 122 (49%) were from Gumarey, and of these, 25 (20%, 95% CI 14.0–29.2) were seropositive, and 126 (51%) were from Sogan-Godud, and of these, 8 (6%, 95% CI 2.7–11.8) were seropositive (Figure 2). Of all samples, 118 (47.6%) were from children <15 years of age, and 4 of the 118 (3.4%) were seropositive. These 4 youngest seropositive participants were 4, 12, 13, and 14 years of age, and all were long-term permanent residents of the study area. Of the 130 adults in the sampled cohort, 29 (22.3%) had positive anti-RVFV IgG results; the oldest was 81 years of age.

### Links between Past Exposure and Seropositivity

Many exposures, both nonanimal and animal, were associated with RVFV seropositivity (Appendix Table 1). In bivariate statistical analyses, RVFV seropositivity varied significantly according to the following factors: age (participants >15 years of age were more at risk, p = 0.0001), gender (male participants were more at risk, p = 0.011), location (those from Gumarey were more at risk, p = 0.001), home flooding (p = 0.024); contact with a dead human body (p = 0.0001); contact with cattle (p = 0.012); and involvement in sheltering (p = 0.003), butchering (p = 0.0001), skinning (p = 0.0001), cooking (p = 0.005), milking (p = 0.0001), birthing livestock (p = 0.0001), or disposing of an aborted animal fetus (p = 0.0001).

**Figure 3:**
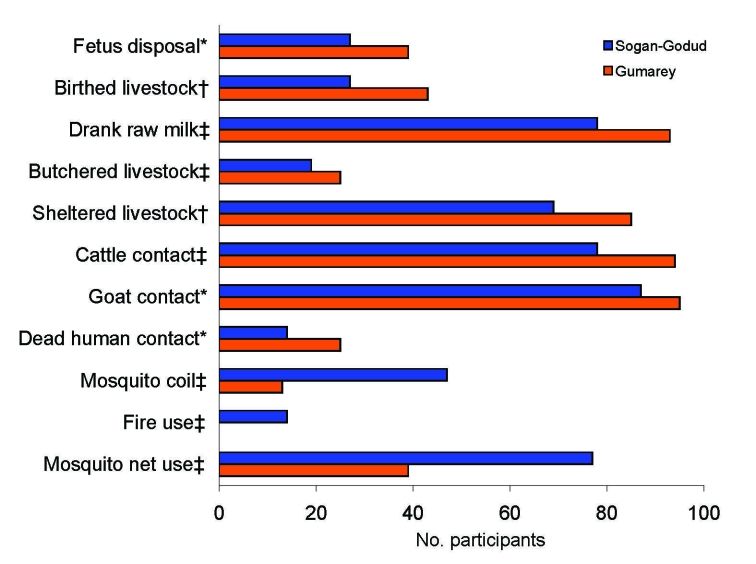
Exposures between northeastern Kenyan villages differed; Gumarey had more animal exposure and Sogan-Godud had more mosquito control. *p<0.05; †p<0.01; ‡p<0.001 (by χ^2^ test).

Other reported exposures varied significantly between the 2 sublocation groups. Those from Sogan-Godud were more likely to have used mosquito nets (odds ratio [OR] 5.2, p = 0.0001) and mosquito coils (OR 8.2, p = 0.0001) to reduce insect exposure. Those from Gumarey were more likely to have had goat contact (OR 2.6, p = 0.046), had cattle contact (OR 4.7, p = 0.0001), consumed raw milk (OR 4.1, p = 0.0001), sheltered livestock (OR 2.6, p<0.002), butchered livestock (OR 1.5, p = 0.0001), birthed livestock in the home (OR 2.1, p = 0.005), disposed of a livestock fetus (OR 1.7, p = 0.005), or to have had direct contact with human remains (OR 2.1, p = 0.026) (Figure 3; Appendix Table 2).

The final logistic model to predict RVFV seropositivity included age, location, gender, and disposal of an aborted animal fetus (Technical Appendix 2). In multivariable logistic regression models used to predict adjusted odds of RVFV seropositivity, location was significant when age and gender were controlled for; those residing in Gumarey were at 4 times the risk of those in Sogan-Godud (adjusted OR 4.15, 95% CI 1.59–10.87). Seropositivity also varied by gender when age and location were controlled for; male participants had >3 times the risk of women participants (20% vs. 9%; adjusted OR 2.78, 95% CI 1.18– 6.58 for male participants vs. female participants), but this difference did not remain significant within sublocation analysis. After age, gender, and location were controlled for, those who had disposed of an aborted animal fetus, were 3 times more likely to be seropositive (72.7% vs. 35.7%, adjusted OR 2.78, 95% CI 1.03–7.52). Age and location, but not gender, were associated with disposal of an aborted animal fetus, such that those who were older or who were from Gumarey were more likely to dispose of an abortus (Technical Appendix 2).

Subgroup analysis by village showed significant predictors of RVFV seropositivity in Gumarey to be an ill family member, disposal of an aborted fetus, and gender (Technical Appendix 2). Displacement by flood was also associated with RVFV seropositivity in Gumarey but could not be included in the model because every seropositive participant was displaced by floods and this factor was overdetermined. Male participants were >3 times as likely to be seropositive compared with female participants: (adjusted OR 3.45, 95% CI 1.17–10.19). Disposal of an aborted animal fetus (adjusted OR 15.12, 95% CI 4.445–51.35) and presence of an ill family member (adjusted OR 18, 95% CI 1.35–246.97) were also associated with RVFV seropositivity. In Sogan-Godud, the logistic model to predict seropositivity included age, such that the odds of seropositivity increased 5% for every 1-year increase in age (adjusted OR 1.05, 95% CI 1.019–1.091) (Technical Appendix 2).

**Figure 4:**
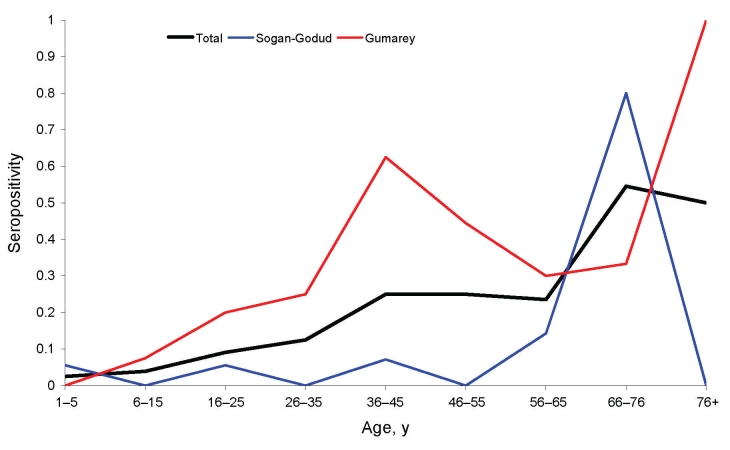
Rift Valley fever virus immunoglobulin G seropositivity by decade of age and village of residence; Gumarey had a higher rate than Sogan-Godud in almost all age groups.

Children <15 years of age had a much lower risk for RVFV seropositivity than those >15 years of age. The adjusted OR for seropositivity (calculated from the overall logistic model) was 1.05; 95% CI 1.03–1.07 per year of age (Figure 4). This difference persisted at the sublocation level with those children in Sogan-Godud still with significantly lower risk than adults.

### Symptom History and Physical Examination Findings

Symptoms and signs reported on the survey questionnaire included fever, malaise, myalgia, chills, backache, eye pain, headache, rash, red eyes, photophobia, poor appetite, flushing, nausea, vomiting, meningismus, poor vision, epistaxis, hematemesis, hematochezia, bruising, confusion, vertigo, stupor, and coma. Of these, a past history of myalgias (OR 6.03, p = 0.0001), backache (OR 3.86, p = 0.003), eye pain (OR 2.28, p = 0.034), red eyes (OR 2.75, p = 0.008), meningismus (OR 2.97, p = 0.004), poor vision (OR 2.74, p = 0.008), and coma (OR 14.55, p = 0.005) were statistically associated with RVFV seropositivity in the study population (Appendix Table 4). Upon physical examination, no nonocular finding was specifically associated with RVFV seropositivity.

### Ophthalmologic Findings

Of the 18 identified cases of substantial retinal disease in the survey population, 7/18 (38.9%) were seropositive compared with 11/18 (61.1%) who were seronegative (p = 0.003, χ^2^ 8.75). All participants with eye disease were >21 years of age, and all seropositive participants with eye disease were >50 years of age. The OR of late eye disease associated with RVFV exposure (seropositivity) was 4.99 (p = 0.003) (Appendix Table 5). Measured visual acuity ranged from 6/5 to 6/60 (equivalent to 20/17–20/200) in the seronegative group and 6/5–6/36 (20/17–20/120) in the seropositive group, although both groups included those with extremely poor vision who could decipher only large objects (measured by finger counting) or who could not perceive light. Visual acuity differed statistically among groups and was more likely to be worse in the RVFV–seropositive group (visual impairment defined as >20/80: 12% of seronegative vs. 25% of seropositive participants; p = 0.047, χ^2^ 3.94). Among the 18 participants with retinal disease, 14 (78%) had visual impairment, and among the 7 seropositive participants with retinal disease, 5 (71%) had visual impairment. No distinctive lesion was associated with RVFV seropositivity, though the eye diseases differed among the groups. Seropositive participants with eye disease had of optic atrophy (3), retinal hemorrhage (2), and retinal scarring (3). One person had retinal hemorrhage and scarring. By contrast, seronegative participants with eye disease had uveitis (1), vasculitis (1), maculopathy (3), peripapillitis (1), retinal scarring (1), optic scarring (2), retinal atrophy (1), and retinal degeneration (1).

## Discussion

This study highlights the variability in RVFV seroprevalence in high-risk settings. In northeastern Kenya, older age, rural village location, male gender, disposal of an aborted fetus, and eye disease were associated with RVFV seropositivity. RVFV seropositivity was relatively high in our sample population in Masalani town, Kenya, particularly in the village area (Gumarey), where seropositivity rates were nearly 4 times higher than in the town area (Sogan-Godud); these areas were separated by only 500 m. Clues to the reasons for this discrepancy in seroprevalence were identified in our study. Those from Gumarey were more likely to have mosquito and animal exposures than those from Sogan-Godud. These risk factors, coupled with the most important predictors of rural seropositivity, male gender, and disposal of an aborted animal fetus, yield evidence for disparate risks for RVFV infection in different communities.

As identified in our prior work, RVFV seroprevalence can vary significantly across Kenya (*16*). Our current study shows that large seroprevalence discrepancies can also occur over short distances. Spatial risk assessments of RVF in animals in Senegal have been predicted by using measurements of seasonal rainfall, land surface temperature, distance to perennial water bodies, and time of year (*18*). Designing such risk maps with human risk factor data may enable improved surveillance systems and better prediction of the spatial distribution of RVFV. This information, gathered with satellite imagery (*19*) and large-scale cluster analysis (*20*), can be used not only to predict large outbreaks but also to identify local hot spots of RVFV transmission to optimize RVF control in resource-limited settings.

For each year of life, the odds of being RVFV seropositive increased by 5%. Male participants were nearly 3 times more likely to be seropositive than female participants, a risk that was noted in the 1997 RVF outbreak investigation (*13*). The difference in seropositivity among genders is not explained on the basis of reported animal or nonanimal exposures, which were comparable and not statistically different between genders. The increased seropositivity among male participants may have a biological basis, given that outcome of infection and resultant immune response to other viruses have been linked to gender differences (*21*).

Disposal of an aborted animal fetus was associated with nearly 3 times increased odds of RVFV seropositivity. This finding may indicate the importance of RVFV transmission by aerosolization of blood and amniotic fluid during animal birthing. It is unknown whether aerosol or vector-borne transmission is the dominant form of transmission during interepidemic or epidemic periods. Our analysis indicates that disposal of an aborted animal fetus was a common associated risk factor at both the composite and sublocation level. Planned repeat sampling of our cohort since the most recent outbreak of 2006–2007 may enable the determination of the primary mode of epidemic transmission.

We found evidence of interepidemic human transmission of RVFV, which has not been previously shown. Our validation of seropositive young children, born after the documented outbreak in 1997–1998, indicate that low-level interepidemic transmission to humans is continuing in the Masalani area and likely in other areas of Kenya (*16*). The natural reservoir for RVFV and the mechanism by which humans become infected during interepidemic periods are unknown. Wild animals have been shown to be infected with RVFV, but further studies must determine whether these animals play a role in RVFV maintenance between outbreaks (*22*).

We demonstrated statistically significant differences between the seropositivity rates of those with and without eye disease. Those with chronic retinal disease were 5 times more likely to be RVFV seropositive. We did observe a difference in visual acuity between RVFV seropositive and seronegative persons in our sample tested 8 years after the 1997–1998 outbreak, and perhaps greater changes may have been present during acute RVF disease. Although there were no ocular findings that were pathognomonic for prior RVFV infection, the detected retinal disease supports evidence from previous studies on the oculopathogenesis of RVFV (*23*).

No specific nonocular examination finding was associated with RVFV seropositivity, but several reported symptoms were statistically more common among those who were RVFV seropositive. Most of these symptoms were severe neurologic manifestations of disease, such as neck stiffness, confusion, and coma. RVFV can cause encephalitis (*1*), and this type of inflammation may explain the higher prevalence of these reported symptoms among seropositive participants. Myalgia and backache may be present in most of the nonsevere RVF cases and are not specific to RVFV infection. Poor vision, which was noted to be more common among RVFV seropositive participants in our sample, may be an indicator for RVF retinitis, a common sequela of RVFV infection (*23*,*24*).

RVFV IgG ELISA and PRNT antibodies are believed to last decades after infection and therefore provide a reliable index of prior RVFV exposure. In contrast, though less well studied, it appears that IgM is lost in 50% of patients after 45 days and is absent in 100% by 4 months after infection (*25*). We did not perform IgM testing in our study, although it might have yielded useful additional information about acute RVFV infection. We also recognize that seropositive results may be false positive due to cross-immunoreactivity with viruses in the same family, although discrepancies between the neutralization test and the ELISA were only 4.9% in this population. The use of confirmatory PRNT testing of ELISA-positive samples can greatly improve viral specificity (*26*).

Our study was limited by its cross-sectional design; therefore, we are unable to conclude whether the identified risk factors specifically caused RVFV exposure. The validity of the associations in this study relies on accurate recall of exposures by the study participants. Although we asked about timing of symptoms and exposures, language differences during questioning limited our accurate collection of these data. Our study may have limited generalizability; we tested risk factors from a small population in Masalani, and risks may vary in other parts of Kenya or in other countries. Data on animal exposures were collected in a binary fashion, so no information about magnitude or duration of contact is known, which may have an effect on risk estimations. We also had no quantitative exposure data for the RVFV vectors in our study area.

This study highlights the large-scale variability in exposure and RVFV seropositivity among Kenyan villages and emphasizes the effect of age, gender, location, and animal husbandry in RVFV transmission. This information is useful for local public health agencies so that they can target protective interventions according to risk factors in different populations. Further studies are needed to examine the epidemiologic, biological, and genetic basis for the increased risk among persons of male gender and to quantify the potential public health impact of modifying the rural environment. RVFV transmission is known to be ongoing in livestock in areas where RVFV is endemic during interepidemic periods; we have shown that this extends to humans, confirming past observations (*27*). Ongoing efforts to predict hot spots of infection on both small and large scales is useful only when at-risk communities are able to use the information to target mosquito or vaccine control efforts and prevent outbreaks. As RVF expands its geographic range and becomes recognized as a disease of global importance for human and animal health, more research is needed to define the most accessible modes of transmission control.

## Supplementary Material

Technical Appendix 1 Study participants received the following structured interview regarding housing, animal exposure, motor function, visual function, and recent or remote Rift Valley fever-related symptoms.

Technical Appendix 2 Binary Logistic Regression Analysis to Predict Rift Valley Fever Virus seropositivity

Appendix Table 1 Associations of potential predictors with Rift Valley fever virus seropositivity*

Appendix Table 2 Association between (village) location and potential model predictors for Rift Valley Fever virus seropositivity*

Appendix Table 3 Testing of association between predictors of Rift Valley fever seropositivity*

Appendix Table 4 Association of signs and symptoms with Rift Valley fever virus seropositivity*

Appendix Table 5 Testing of association of eye disease with Rift Valley fever virus seropositivity
