# Supplementary material for: Interepidemic Rift Valley Fever Virus Seropositivity, Northeastern Kenya
Source: Emerg Infect Dis. 2008 Aug;14(8):1240–6. doi: 10.3201/eid1408.080082 (PMC2600406; doi:10.3201/eid1408.080082)
Supplement: Technical Appendix 1 — Study participants received the following structured interview regarding housing, animal exposure, motor function, visual function, and recent or remote Rift Valley fever-related symptoms. [file 08-0082_Techapp1-s1.pdf]

Interepidemic Rift Valley Fever Virus Seropositivity, Northeastern Kenya

Technical Appendix 1

Study participants received the following structured interview regarding housing, animal exposure, motor function, visual function, and recent or remote Rift Valley fever–related symptoms.

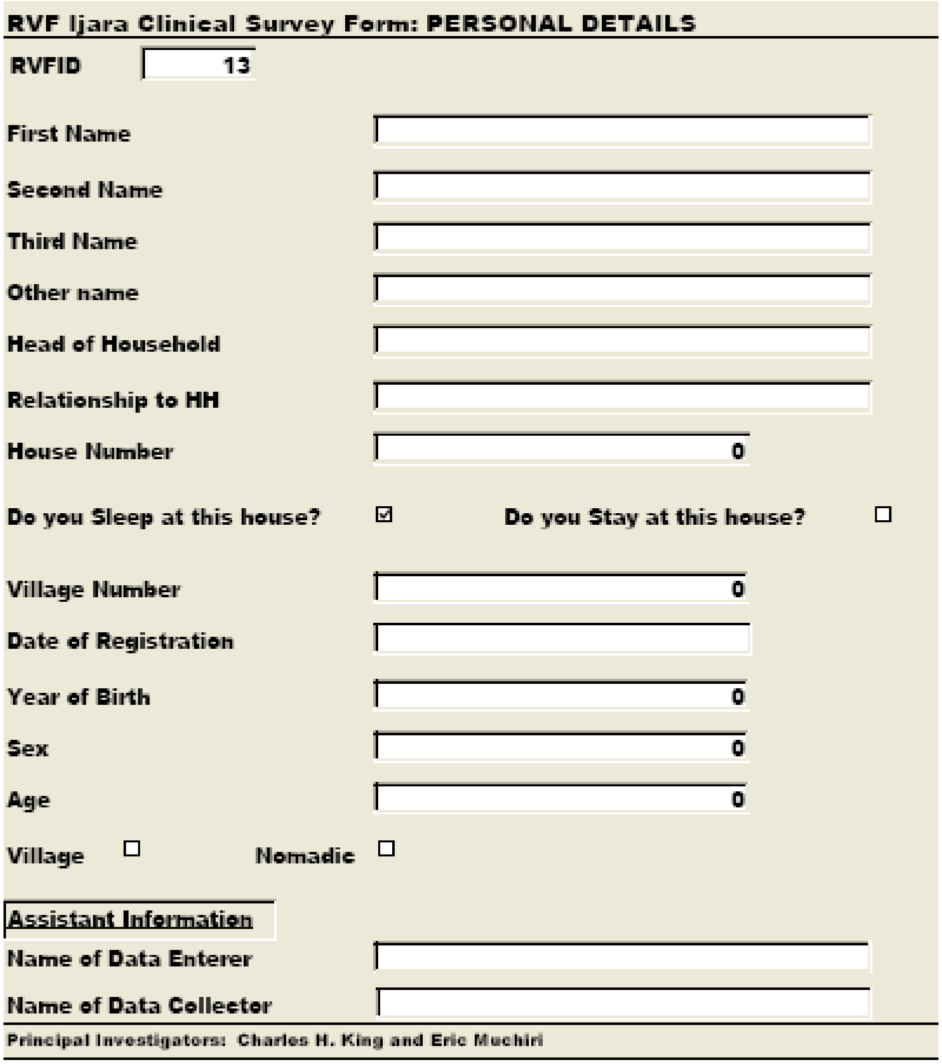
RVF Ijara Clinical Survey Form: PERSONAL DETAILS

RVFID 13

First Name

Second Name

Third Name

Other name

Head of Household

Relationship to HH

House Number 0

Do you Sleep at this house? ☑ Do you Stay at this house? ☐

Village Number 0

Date of Registration

Year of Birth 0

Sex 0

Age 0

Village ☐ Nomadic ☐

Assistant Information

Name of Data Enterer

Name of Data Collector

Principal Investigators: Charles H. King and Eric Muchiri

RVF Ijara Clinical Survey Form: EXPOSURES

RVFID 11000

Data Collector Name

Data Enterer Name

Non-Animal Exposures

What type of settlement do you live in?

Was your home ever flooded? ☐

When was it flooded?

Have you ever been displaced by a flood? ☐

When were you displaced?

Do you use a mosquito net? ☐

How often do you use the net?

Do you use fire? ☐

How often do you use fire?

Do you use other forms of mosquito control?

Have you had a recent mosquito bite? ☐

Have you had any personal illness? ☐

When were you ill?

Have you had an ill family member? ☐

When was your family member ill?

Have you had contact with a dead human body? ☐

When was your contact with a dead body?

Animal Exposures

Please check any animal contact. Sheep contact ☐ Cow contact ☐ Goat contact ☐ Camel contact ☐

Have you sheltered livestock in your home? ☐

camel ○ sheep ○ goat ○ cow ○ Other

Have you killed an animal? ☐

camel ○ sheep ○ goat ○ cow ○ Other

Have you butchered an animal? ☐

camel ○ sheep ○ goat ○ cow ○ Other ______

Have you skinned an animal? ☐

camel ○ sheep ○ goat ○ cow ○ Other ______

Have you cooked with meat? ☐

camel ○ sheep ○ goat ○ cow ○ Other ______

Have you milked an animal? ☐

camel ○ sheep ○ goat ○ cow ○ Other ______

Have you ever drank raw animal milk? ☐

camel ○ sheep ○ goat ○ cow ○ Other ______

Have you ever cared for birthing animal? ☐

camel ○ sheep ○ goat ○ cow ○ Other ______

Have you ever disposed of an aborted animal fetus? ☐

camel ○ sheep ○ goat ○ cow ○ Other ______

Symptoms Have you ever had any of the following symptoms?
If yes, please indicate when.

Fever	☐		Red eyes	☐		Hard to arouse	☐	
Sick Feeling	☐		No appetite	☐		Coma	☐	
Muscle aches	☐		Flushing	☐		Neck stiffness	☐	
Chills	☐		Nausea	☐		Poor vision	☐	
Backache	☐		Vomiting	☐		Nosebleeds	☐	
Eye pain	☐		Painful eyes to light	☐		Vomiting Blood	☐	
Headache	☐		Confusion	☐		Bloody stool	☐	
Rash	☐		Spinning	☐		Bruising	☐	

Principal Investigators: Charles H. King and Eric Muchiri

RVF Ijara Clinical Survey Form: PHYSICAL EXAM

RVFID 0 Weight 0 Height 0

General Wasted ☐

Head

Eyes

Scleral hemorrhages ☑ Scleral Icterus ☐

Ears

Nose

Throat

Neck

Normal movement ☐

Chest

Heart

Murmur ☐

Abdomen

Hepatomegaly ☐ Splenomegaly ☐

GU

Neuro

Skin

Jaundice ☐ Petechiae ☐ Purpura ☐ Ecchymosis ☐

Lymphadenopathy

Cervical ☐ Axillary ☐ Inguinal ☐

Other

Name of Medical Doctor

Name of Data Enterer

Principal Investigators: Charles H. King and Eric Muchiri

RVF Ijara Clinical Survey Form: OPHTHALMOLOGIC EXAM

RVFID: 0

Visual Acuity-OS: 0

Visual Acuity-OD: 0

Anterior Chamber-OS:

Anterior Uveitis-OS ☐

Anterior Chamber-OD:

Anterior Uveitis-OD ☐

Posterior Chamber-OS:

Vitreous reaction-OS ☐

Posterior Chamber-OD:

Vitreous reaction-OD ☑

Retina-OS:

Retinitis-OS ☐ Macular-OS ○ Paramacular-OS ○

Retinal Hemorrhage-OS ☐

Zone-OS:

Area-OS:

Optic disc edema-OS ☐

Retinal vasculitis-OS ☐

Retina-OD:

Retinitis-OD ☐ Macular-OD ○ Paramacular-OD ○

Retinal Hemorrhage-OD ☐

Zone-OD: 0

Area-OD:

Optic disc edema-OD ☐

Retinal vasculitis-OD ☐

RVF Related Disease-OS ☐

RVF Related Disease-OD ☐

Comments:

Ophthalmologist Name:

Data Enterer Name:

Principal Investigators: Charles H. King and Eric Muchiri
