# Supplementary material for: Interepidemic Rift Valley Fever Virus Seropositivity, Northeastern Kenya
Source: Emerg Infect Dis. 2008 Aug;14(8):1240–6. doi: 10.3201/eid1408.080082 (PMC2600406; doi:10.3201/eid1408.080082)
Supplement: Technical Appendix 2 — Binary Logistic Regression Analysis to Predict Rift Valley Fever Virus seropositivity [file 08-0082_Techapp2-s2.pdf]

Interepidemic Rift Valley Fever Virus Seropositivity, Northeastern Kenya

Technical Appendix 2

Binary Logistic Regression Analysis to Predict Rift Valley Fever Virus seropositivity

Logistic Model 1

Outcome variable: Rift Valley fever seropositivity (coded as 0 vs. 1)*

Predictor variable	Variable type	Point estimate (CI)	p value
Age	Continuous	1.039 (1.017–1.062)	0.001
Location (Sogan-Godud vs. Gumarey)	Dichotomous	0.241 (0.092–0.628)	0.004
Gender (male vs. female)	Dichotomous	2.782 (1.176–6.581)	0.020
Disposal of aborted animal	Dichotomous	2.779 (1.026–7.525)	0.044
Constant		0.119	0.005

*CI, confidence interval. Goodness-of-fit: χ^2 analysis of observed vs. predicted values indicating model is well fit to observed values. Hosmer and Lemeshow test, step 1: χ^2, 8.813; degrees of freedom, 8; p value, 0.358.

Logistic Model by Location, Gumarey

Outcome variable: Rift Valley fever seropositivity*

Predictor variables	Variable type	Point estimate (CI)	p value
Gender	Dichotomous	3.454 (1.17–10.19)	0.025
Discarded aborted animal fetus	Dichotomous	15.12 (4.45–51.35)	0.0001
Ill family member	Dichotomous	18 (1.35–246.97)	0.029
Constant		0.029	0.0001

* CI, confidence interval. Goodness-of-fit: χ^2analysis of observed vs. predicted values indicating model is well fit to observed values. Hosmer and Lemeshow test, step 1: χ^2, 5.493; degrees of freedom, 2; p value, 0.064.

Logistic Model by Location, Sogan-Godud

Outcome variable: Rift Valley fever seropositivity

Predictor variables	Variable type	Point estimate (CI)	p value
Age	Continuous	1.054 (1.019–1.091)	0.0001
Constant		0.01	0.0001

* CI, confidence interval. Goodness-of-fit: χ^2analysis of observed vs. predicted values indicating model is well fit to observed values. Hosmer and Lemeshow test, step 1: χ^2, 9.318; degrees of freedom, 7; p value, 0.231.
