# Supplementary material for: Interepidemic Rift Valley Fever Virus Seropositivity, Northeastern Kenya
Source: Emerg Infect Dis. 2008 Aug;14(8):1240–6. doi: 10.3201/eid1408.080082 (PMC2600406; doi:10.3201/eid1408.080082)
Supplement: Appendix Table 1 — Associations of potential predictors with Rift Valley fever virus seropositivity* [file 08-0082_appT1-s3.pdf]

Appendix Table 1. Associations of potential predictors with Rift Valley fever virus seropositivity*

Variable	Test statistic	p value‡	Odds ratio
Age (continuous)	6.184 (Mean difference = 22.6)	0.0001	
Age (>15 y vs. 1–14 y)	18.772	0.0001	8.032
Location (Gumarey vs. Sogan-Godud)	10.747	0.001	3.8
Gender (Male vs. female)	6.546	0.011	2.59
Home flooded	5.105	0.024	
Displacement by flood	2.696	0.101	
Contact with dead human body	36.97	0.0001	
Use of mosquito nets	2.655	0.103	
Use of fire	0.038	0.864	
Mosquito coils	3.6	0.058	0.392
Recent mosquito bite	0.038	0.538	
Recent illness	3.045	0.081	
Ill family member	0.154	0.43	
Sheep contact	2.22	0.136	
Goat contact	2.39	0.122	
Camel contact	4.75	0.029	
Cattle contact	6.288	0.012	
Sheltering	8.623	0.003	
Slaughtering	3.11	0.078	
Butchering	23.817	0.0001	
Skinning	15.237	0.0001	
Cooking	7.878	0.005	
Milking	16.64	0.0001	
Birthing livestock	32.447	0.0001	
Disposal of animal fetus	28.303	0.0001	
Drinking raw animal milk	3.077	0.079	

*All variables were dichotomous except age (continuous).
†Pearson χ^2 test with Yates continuity correction was used for all variables except age (continuous), which used independent samples 2-tailed *t* test.
‡p<0.05 was statistically significant.
