# Supplementary material for: Interepidemic Rift Valley Fever Virus Seropositivity, Northeastern Kenya
Source: Emerg Infect Dis. 2008 Aug;14(8):1240–6. doi: 10.3201/eid1408.080082 (PMC2600406; doi:10.3201/eid1408.080082)
Supplement: Appendix Table 2 — Association between (village) location and potential model predictors for Rift Valley Fever virus seropositivity* [file 08-0082_appT2-s4.pdf]

Appendix Table 2. Association between (village) location and potential model predictors for Rift Valley Fever virus seropositivity*

Variable	Test statistic†	p value‡	Odds ratio (Gumarey vs. Sogan-Godud)
Age (continuous)	0.161	0.873	
Age (>15 y vs. 1–14 y)	1.278	0.258	0.75
Location (Gumarey vs. Sogan-Godud)	10.747	0 = 0.001	3.8
Gender (Male vs. female)	0.727	0.394	1.25
Home flooded	0.57	0.45	0.79
Displacement by flood	0.194	0.66	0.876
Contact with dead human body	4.9	0.026	2.09
Use of mosquito nets	36.16	0.0001	0.194
Use of fire	17.67	0.0001	NA
Mosquito coils	41.75	0.0001	0.122
Recent mosquito bite	0.263	0.61	0.802
Recent illness	0.081	0.776	1.075
Ill family member	3.242	0.072	0.354
Sheep contact	3.27	0.07	2.43
Goat contact	3.98	0.046	2.64
Cattle contact	14.09	0.0001	4.74
Camel contact	4.498	0.034	0.503
Sheltering	9.41	0.002	2.62
Slaughtering	2.45	0.117	6.4
Butchering	1.776	0.0001	1.5
Skinning	0.875	0.35	1.28
Cooking	1.812	0.178	1.44
Milking	0.039	0.843	1.05
Birthing livestock	7.9	0.005	2.14
Disposal of aborted animal fetus	4.15	0.042	1.74
Drinking raw animal milk	12.45	0.0001	4.12

*All variables were dichotomous except age (continuous).
†Pearson χ^2 test with Yates continuity correction was used for all variables except age (continuous), which used independent samples 2-tailed *t* test.
‡p<0.05 was statistically significant.
