# Supplementary material for: Interepidemic Rift Valley Fever Virus Seropositivity, Northeastern Kenya
Source: Emerg Infect Dis. 2008 Aug;14(8):1240–6. doi: 10.3201/eid1408.080082 (PMC2600406; doi:10.3201/eid1408.080082)
Supplement: Appendix Table 3 — Testing of association between predictors of Rift Valley fever seropositivity* [file 08-0082_appT3-s5.pdf]

Appendix Table 3. Testing of association between predictors of Rift Valley fever seropositivity*

Age	Age (p value)	Village (p value)	Gender (p value)
Village	0.161 (0.873)		
Gender	0.151 (0.886)	0.727 (0.394)	
Disposal of aborted animal fetus	10.25 (0.0001)	4.145 (0.042)	0.278 (0.598)

*Testing for interaction between associated predictors: (Disposal of aborted animal fetus) × Age; (Disposal of aborted animal fetus) × Village. Both interaction terms were not significant (p>0.05). The goodness-of-fit statistic indicates that the additional interaction terms were not beneficial to the predictive power of the model.
