# Supplementary material for: Interepidemic Rift Valley Fever Virus Seropositivity, Northeastern Kenya
Source: Emerg Infect Dis. 2008 Aug;14(8):1240–6. doi: 10.3201/eid1408.080082 (PMC2600406; doi:10.3201/eid1408.080082)
Supplement: Appendix Table 4 — Association of signs and symptoms with Rift Valley fever virus seropositivity* [file 08-0082_appT4-s6.pdf]

Appendix Table 4. Association of signs and symptoms with Rift Valley fever virus seropositivity*

Variable	Test statistic†	p value‡	Odds ratio
Personal illness	3.045	0.081	
Family illness	0.154	0.43	
Fever	0.109	0.741	
Malaise	1.77	0.183	
Myalgia	12.97	0.0001	6.03
Chills	2.962	0.085	
Backache	9.059	0.003	3.864
Eye pain	4.511	0.034	2.275
Headache	0.035	0.852	
Rash	0.134	0.714	
Red eyes	7.036	0.008	2.75
Photophobia	3.63	0.057	
Poor appetite	0.504	0.478	
Flushing	0.752	0.386	
Nausea	0.373	0.541	
Vomiting	1.36	0.243	
Meningismus	8.24	0.004	2.97
Poor vision	6.985	0.008	2.74
Epistaxis	0.181	0.67	
Hematemesis	2.52	0.112	
Hematochezia	0.372	0.542	
Bruising	1.054	0.305	
Confusion	0.366	0.551	
Vertigo	0.092	0.761	
Stupor	1.173	0.279	
Coma	7.93	0.005	14.55

*All variables were dichotomous.
†Pearson χ^2 test with Yates continuity correction was used for all variables.
‡$p<0.05$ was statistically significant.
