# Supplementary material for: Interepidemic Rift Valley Fever Virus Seropositivity, Northeastern Kenya
Source: Emerg Infect Dis. 2008 Aug;14(8):1240–6. doi: 10.3201/eid1408.080082 (PMC2600406; doi:10.3201/eid1408.080082)
Supplement: Appendix Table 5 — Testing of association of eye disease with Rift Valley fever virus seropositivity [file 08-0082_appT5-s7.pdf]

Appendix Table 5. Testing of association of eye disease with Rift Valley fever virus seropositivity

Variable	Test statistic*	p value†	Odds ratio
Eye disease	11.011	0.003	4.99

*Pearson χ_2 test with Yates continuity correction.
†$p<0.05$ was statistically significant.
